# Comparing the Rat Grimace Scale and a composite behaviour score in rats

**DOI:** 10.1371/journal.pone.0209467

**Published:** 2019-05-31

**Authors:** Cassandra B. Klune, Amy E. Larkin, Vivian S. Y. Leung, Daniel Pang

**Affiliations:** 1 David Geffen School of Medicine, University of California Los Angeles, Los Angeles, California, United States of America; 2 Department of Small Animal Clinical Sciences, Western College of Veterinary Medicine, Saskatoon, Saskatchewan, Canada; 3 Faculty of Veterinary Medicine, Université de Montréal, Saint-Hyacinthe, Québec, Canada; University of Bari, ITALY

## Abstract

There is a growing interest in the use of voluntarily displayed ongoing behaviours in laboratory animals to assess the pain experience. In rats, two behavioural pain scales, the Rat Grimace Scale (RGS, a facial expression scale) and a composite behaviour score (CBS, a behavioural ethogram reliant on postural changes), are both promising pain assessment methods. Both scales have been used to assess pain in a laparotomy model, however, they have never been compared directly and the knowledge of how different analgesics may affect these two scales is limited. This study aimed to provide a comparison to discriminate the temporal and analgesic response in a laparotomy model. Female Wistar (n = 26) and Sprague Dawley rats (n = 26) were block randomized to receive saline, meloxicam (2 mg/kg) or buprenorphine (0.05 mg/kg) 30 minutes before laparotomy. Rats were video-recorded before surgery (BL) and at 30, 150, 270, and 390 minutes post-operatively. Videos were assessed according to both scales by a trained, blinded observer. Both CBS and RGS scores increased significantly at all post surgical timepoints in the saline group. Both buprenorphine and meloxicam reduced CBS scores to baseline levels following laparotomy; however, RGS scores were only reduced following buprenorphine. RGS scores in the meloxicam group remained similar to scores of the saline group. These findings suggest that the CBS and RGS differ in their sensitivity to discriminating analgesic effects.

## Introduction

Accurate and reliable pain assessment in laboratory rodents is essential to produce high quality pain research and safeguard animal welfare. The continued dependence on evoked hypersensitivity to assess pain in animals has been proposed as a contributor towards failure of translational research. While measures of evoked hypersensitivity assess hyperalgesia and allodynia, they do not capture ongoing pain, which has been suggested to be most relevant in many human pain conditions [1–5]. Ongoing pain is perpetuated by an ongoing inflammatory process rather than an external stimulus. Multiple methods have been proposed to evaluate ongoing pain in animals; however, a lack of evidence describing the strengths and weaknesses of such methods in comparison to one another discourages their use [3].

From a welfare perspective, signs associated with pain are common humane endpoints in rodent research, but typical signs such as weight loss may not be specific to pain or sufficiently sensitive to be useful [6]. Traditional nociceptive tests, such as mechanical withdrawal testing, are not used as welfare assessment tools as they are time consuming and labour intensive.

Two promising behavioural assessment methods have been developed to better capture the ongoing pain experience of laboratory rodents. These are the Rat Grimace Scale (RGS) [7] and the short form Composite Behaviour Score (CBS) [8–10]. The RGS, a facial expression scale, measures the degree of change in four ‘action units’: orbital tightening, nose/cheek flattening, ear, and whisker changes. The CBS consists of counting the frequency of select full body behaviours that have been associated with pain (i.e., writhing, back arching and staggering).

Both scales are responsive to predicted changes in pain levels (increasing after a painful intervention and decreasing following analgesic administration) and have been shown to have good construct validity and inter-rater reliability [7–12]. Both scales are also relatively simple to employ. However, the independent use of these scales has provided conflicting reports on the efficacy of commonly used analgesics in reducing pain scores. CBS scores were repeatedly shown to decrease with administration of non-steroidal anti-inflammatory drugs (NSAIDs) [8–10]. In contrast, RGS scores only decreased with some NSAIDs or if used at high doses [13]. This suggests that these scales may not be equally sensitive in discriminating analgesic response.

The aim of this study was to compare the performance of the CBS and RGS in a laparotomy model of acute post-operative pain, using meloxicam and buprenorphine, two commonly used analgesics of different drug classes. A secondary aim was to evaluate any effects of rat strain on scale by using both Wistar and Sprague-Dawley rats. It was hypothesized that scores from both scales would decrease with analgesia and that animals treated with buprenorphine would display lower pain scores compared to those treated with meloxicam. Rat strain was not hypothesized to have an effect on pain scores or analgesic response.

## Materials and methods

All experiments were approved by the University of Calgary Health Sciences Animal Care Committee, Calgary, Canada (protocol ID: AC15-0062), in accordance with Canadian Council on Animal Care guidelines.

### Animals

Adult female Wistar (n = 26) and Sprague Dawley rats (n = 27) that were at least 6 weeks of age were obtained from Charles River, Canada (250 ± 100g) or surplus stock at the University of Calgary Animal Resource Centre. Plastic cages (47 x 25 x 21cm, RC88D-UD, Alternate Design Mfg and Supply, Siloam Springs, Arizona, USA) with wood chip bedding, shredded paper, and a plastic enrichment tube were used for housing. Rats were pair housed and kept on a 12hr light dark cycle (lights on at 7:00 hours) in a temperature (23°C) and humidity (22%) controlled environment free of pathogens and negative serologically for all antigens tested on the Charles River Assessment plus. Rats were provided food (Prolab 2500 Rodent 5P14, LabDiet, PMI Nutrition International, St. Louis, MO, USA Prolab 2500 Rodent 5P14, LabDiet, PMI Nutrition International, St. Louis, MO, USA) and water *ad libitium*. Before experiments began, rats were habituated for a minimum of three days. Habituation consisted of 10 minutes of handling by each experimenter (CK and AL) and 10 minutes in a plexi-glass observation box (W 14 cm x L 26.5 cm x H 20.5 cm) in which rats would be video recorded. During handling and exposure to the observation box, rats were offered a food reward (Honey Nut Cheerios^TM^, General Mills, Inc., Golden Valley, MN, USA). Rats were block randomized (list randomizer, random.org) to receive either meloxicam (n = 16, 2 mg/kg; Metacam Solution for Injection, Boehringer Ingelheim Vetmedica, Inc., St. Joseph, MO, USA) or saline (n = 16, volume matched to meloxicam). The dose of 2mg/kg was chosen as it has previously been shown to reduce laparotomy pain according to the CBS [9]. Following preliminary analysis, a buprenorphine treatment group was added to act as a positive drug control. A second cohort of rats were randomised to receive saline (n = 5, volume matched to meloxicam) or buprenorphine (n = 16, 0.05 mg/kg; Vetergesic, 0.3 mg/mL; Champion Alstoe, Whitby, ON, Canada). The buprenorphine dose of 0.05mg/kg was chosen as it is within the range reported as effective for treating post-operative pain [14]. All procedures were performed during the light period, between 0730 and 1800.

### Surgery

All surgeries were performed by a single surgeon (CK). Thirty minutes before induction of anesthesia, rats received a subcutaneous injection of either saline, meloxicam, or buprenorphine. Rats were anesthetized in a plexi-glass induction chamber with 2% isoflurane carried in oxygen at 1L/min. Following loss of the righting reflex, rats were removed from the chamber and anaesthesia was maintained using a face mask. Rats were placed in dorsal recumbency on a heating pad (Sunbeam, 50watts, 120VAC, UL, USA) and ocular lubricant was placed in both eyes. The abdomen of the rat was shaved and aseptically prepared using chlorohexidine and 70% alcohol. Rats were then covered with a sterile drape so only the surgical area was exposed. A three-centimeter incision in the skin was made beginning 1 cm caudal to the xyphoid cartilage using a scalpel blade (size 15). A three-centimeter incision was made through the muscle layer by a stab incision lengthened with Metzenbaum scissors. The muscle was then closed using a simple continuous suture pattern and the skin using a subcuticular suture pattern (4–0 Monocryl (Poliglecaprone) Suture, RB-1 17mm Taper, Ethicon). Tissue glue (3M Vetbond, 3 mls, 3M Animal Care Products, St Paul, MN, USA) was used to cover knots that could not be buried in the skin. Following the skin closure, isoflurane was terminated and rats were left to recover with oxygen only. Rectal temperature was taken at this time, before the rats regained sternal recumbency. Once sternal, rats were returned to their home cages, which were warmed using a heating lamp for 150 minutes following surgery. Rectal temperatures were taken at the at 30 minute and 150 minute timepoints after video recording was completed to ensure recovery to normothermia.

### Video recording

Rats were placed individually in the observation box in view of two cameras (Panasonic HC-V720P/PC, Panasonic Canada Inc., Mississauga, ON, Canada) positioned perpendicular to one another (one on the short side of the box and the other on the long side of the box). Rats were recorded for 15 minutes at 45 minutes before surgery (baseline; BL) and 30, 150, 270 and 390 minutes after surgery. All recordings took place in a dedicated behavioural assessment room. After the last recording period, rats were euthanized by first being anesthetised with isoflurane and, following loss of the righting reflex, overdose with carbon dioxide. Death was confirmed with cessation of heart beat.

### Video analysis

#### The Rat Grimace Scale

Observers (CK and AL) blinded to treatment and time point took screenshots of each rat’s face throughout the 15 minute video. Four images were taken per video approximately 3.5 minutes apart but no less than one minute apart. A clear frontal view of the face was captured whenever possible. If this was not possible, a side profile was captured instead. Images were placed into presentation software (Microsoft PowerPoint, version 15.0, Microsoft Corporation, Redmond, WA, USA) and slide order was randomized (http://www.tushar-mehta.com/powerpoint/randomslideshow/index.htm). A trained observer (CK) blinded to treatment and timepoint scored all images using the RGS [15]. Action units (orbital tightening, ear changes, nose/cheek flattening and whiskers changes) were scored as either “0” if not present, ‘1’ if moderately present or “2” if obviously present, according to the original method described by Sotocinal et al. [7]. These action unit scores were averaged to produce a score between 0–2 for each image. Following unblinding of images, scores collected at each time point were averaged for each animal.

#### Composite behaviour score

Trained observers (CK and AL) blinded to treatment and time point watched the first 10 minutes of the 15 minute long video and counted the frequency of each discrete occurrence of three pain behaviours: 1) Back-arch: upward arching of the back in a cat-like behavior 2) Writhe: contraction of flank abdominal muscles producing concavity of the side of the rat caudal to the rib cage 3) Stagger: an occurrence of falling or a quicker than normal movement of the feet characterized by the loss of balance [10]. These behaviours were identified to be the major signs of pain following laparotomy, by Roughan and Flecknell [10] and each occurrence of each behavior counted as one point towards the overall pain score of the rat.

### Statistical analysis

A sample size of 12 animals per treatment group was estimated using an alpha value of 0.05 and a power of 0.8 to detect a mean difference of 0.3 with the RGS [16]. The D’Agostino and Pearson omnibus normality test was used to assess the normality of each data set. Strain differences within each treatment group were tested using two-way ANOVA with a Sidak correction for multiple comparisons. No strain differences were identified so data were pooled for further analysis. A two-way ANOVA with a Greenhouse-Geisser correction and a Tukey correction for multiple comparisons was applied to the RGS and CBS data sets to assess main effects of drug treatment and time. To assess for increases from baseline a two-way ANOVA with Dunnett post-hoc test was applied to analyse each data set. A p-value less than 0.05 was considered significant. Data are presented as mean ± SEM with the 95% confidence interval (95%CI) for the mean difference. Coefficients of variation were calculated for each group at each timepoint. A Spearman’s rank correlation coefficient was used to assess the relationship between RGS and CBS scores. Data were analyzed with commercial software (Prism 6.07, GraphPad Software, La Jolla, CA, USA). Data supporting the results are available in an electronic repository: https://doi.org/10.7910/DVN/CTOVDW.

## Results

Data from one rat were excluded from the buprenorphine (Sprague-Dawley) group due to a mis-injection. Rats were normothermic at the time of sternal recumbency. One rat (Wistar treated with saline) dropped below the normothermic range (35.5–37.7°C, corrected rectal temperature [17]) at 30 minutes after sternal recumbency but was normothermic by the 150 minute timepoint. No other complications following surgery were encountered.

**Fig 1:**
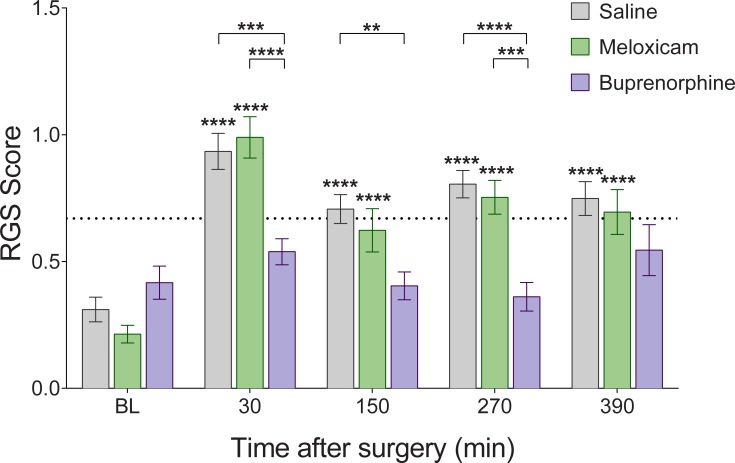
Rat Grimace Scale scores are reduced by the administration of buprenorphine after laparotomy. Saline (n = 21), meloxicam (n = 16) and buprenorphine (n = 15) groups at all time points. Data presented as mean ± SEM. BL = baseline. Asterisks above bars indicate within group differences from baseline. Asterisks with brackets indicate differences between groups. Horizontal broken line at y = 0.67 indicates analgesic intervention threshold [11].

**Fig 2:**
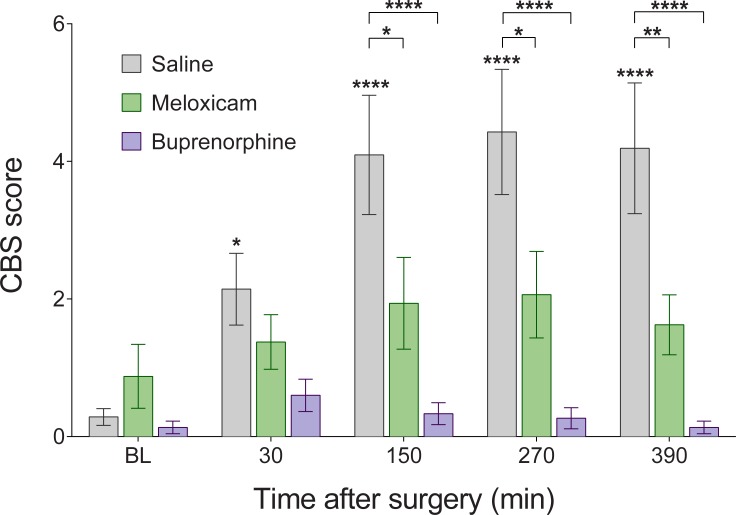
Composite behaviour scores (cumulative frequency of back-arching, writhing and staggering) are reduced by both meloxicam and buprenorphine following laparotomy. Saline (n = 21), meloxicam (n = 16) and buprenorphine (n = 15) groups at all time points. Data presented as mean ± SEM. BL = baseline. Asterisks stars above bars indicate within group differences from baseline. Asterisks with brackets indicate differences between groups.

There were no differences in RGS or CBS scores between the two strains: Wistar and Sprague Dawley rats (p > 0.05; S1 Table). Pain scores within the saline group increased from baseline at all post-surgical timepoints with both the RGS and the CBS (p < 0.05; Table 1, Figs 1 and 2). The RGS and CBS scores of rats treated with buprenorphine remained similar to baseline levels (p > 0.05; Table 1, Figs 1 and 2). The CBS scores of rats treated with meloxicam also remained similar to baseline levels at all post-surgical timepoints (p > 0.05, Fig 2) while, in contrast, the RGS scores for meloxicam treated rats increased significantly from baseline (p < 0.05; Table 1, Fig 1).

**Table 1:** Within group comparisons (to baseline) of RGS and CBS scores at all post-surgical timepoints.

	RGS	CBS
Comparison	p-value [95% CI]	p-value [95% CI]
**Saline**		
BL vs. 30	**< 0.0001** [0.43 to 0.82]	**0.014** [0.30 to 3.4]
BL vs. 150	**< 0.0001** [0.20 to 0.59]	**< 0.0001** [2.3 to 5.4]
BL vs. 270	**< 0.0001** [0.30 to 0.69]	**< 0.0001** [2.6 to 5.7]
BL vs. 390	**< 0.0001** [0.24 to 0.64]	**< 0.0001** [2.3 to 5.5]
**Meloxicam**		
BL vs. 30	**< 0.0001** [0.55 to 1.0]	0.90 [-1.3 to 2.3]
BL vs. 150	**< 0.0001** [0.18 to 0.64]	0.39 [-0.72 to 2.8]
BL vs. 270	**< 0.0001** [0.31 to 0.77]	0.29 [-0.60 to 3.0]
BL vs. 390	**< 0.0001** [0.25 to 0.71]	0.69 [-1.0 to 2.5]
**Buprenorphine**		
BL vs. 30	0.51 [-0.11 to 0.36]	0.93 [-1.4 to 2.3]
BL vs. 150	> 0.99 [-0.25 to 0.22]	> 0.99 [-1.6 to 2.0]
BL vs. 270	0.94 [-0.29 to 0.18]	> 0.99 [-1.7 to 2.0]
BL vs. 390	0.46 [-0.11 to 0.36]	> 0.99 [-1.8 to 1.8]

p-values and 95% confidence intervals of the differences are reported for each comparison.

The RGS scores of saline- and meloxicam-treated animals were similar at all timepoints (p > 0.05; Table 2, Fig 1). Buprenorphine treated rats displayed lower RGS scores than saline treated rats at 30 minutes,150 minutes, and 270 minutes (p < 0.05) but not at baseline or 390 minutes (p > 0.05; Table 2, Fig 1). Buprenorphine treated animals also displayed lower RGS scores than meloxicam treated animals at 30 minutes and 270 minutes (p < 0.05) but not at baseline, 150 minutes, or 390 minutes (p > 0.05; Table 2, Fig 1).

**Table 2:** Between group comparisons of RGS and CBS scores at all timepoints.

	RGS	CBS
Comparison	p-value [95% CI]	p-value [95% CI]
**Saline vs**. **Meloxicam**		
BL	0.55 [-0.12 to 0.31]	0.76 [-2.5 to 1.4]
30 min	0.82 [-0.27 to 0.16]	0.62 [-1.2 to 2.7]
150 min	0.64 [-0.13 to 0.30]	**0.026** [0.21 to 4.1]
270 min	0.84 [-0.17 to 0.27]	**0.013** [0.41 to 4.3]
390 min	0.83 [-0.16 to 0.27]	**0.0061** [0.61 to 4.5]
**Saline vs**. **Buprenorphine**		
BL	0.50 [-0.33 to 0.12]	0.98 [-1.8 to 2.1]
30 min	**0.0001** [0.17 to 0.62]	0.16 [-0.45 to 3.5]
150 min	**0.0042** [0.081 to 0.52]	**< 0.0001** [1.8 to 5.8]
270 min	***<* 0.0001** [0.22 to 0.67]	**< 0.0001** [2.2 to 6.2]
390 min	0.080 [-0.018 to 0.43]	**< 0.0001** [2.1 to 6.0]
**Meloxicam vs**. **Buprenorphine**		
BL	0.11 [-0.44 to 0.033]	0.69 [-1.4 to 2.9]
30 min	**< 0.0001** [0.21 to 0.69]	0.66 [-1.3 to 2.9]
150 min	0.076 [-0.017 to 0.45]	0.18 [-0.51 to 3.7]
270 min	**0.0003** [0.16 to 0.63]	0.11 [-0.32 to 3.9]
390 min	0.29 [-0.086 to 0.39]	0.22 [-0.62 to 3.6]

p-values and 95% confidence intervals of the differences are reported for each comparison.

Meloxicam treated animals displayed CBS scores significantly lower than saline treated animals at 150 minutes, 270 minutes, and 390 minutes (p < 0.05) but not baseline or 30 minutes (p > 0.05; Table 2, Fig 2). Similarly, buprenorphine treated animals also displayed CBS scores lower than saline treated animals at 150 minutes, 270 minutes, and 390 minutes (p < 0.05) but not at baseline or 30 minutes (p > 0.05; Table 2, Fig 2). CBS scores from buprenorphine treated rats did not differ from those of meloxicam treated animals at any timepoint (p > 0.05; Table 2, Fig 2).

**Fig 3:**
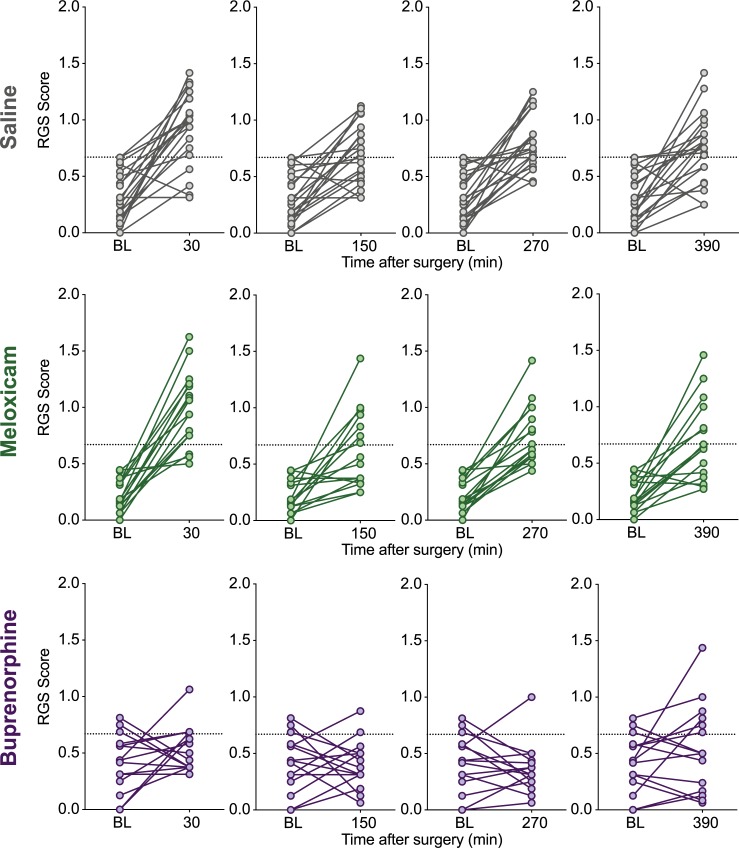
RGS scores of individual rats at all post-surgical timepoints in comparison to their baseline scores. Saline (n = 21), meloxicam (n = 16) and buprenorphine (n = 15) rats at all time points. Horizontal broken line at y = 0.67 indicates analgesic intervention threshold [11].

**Fig 4:**
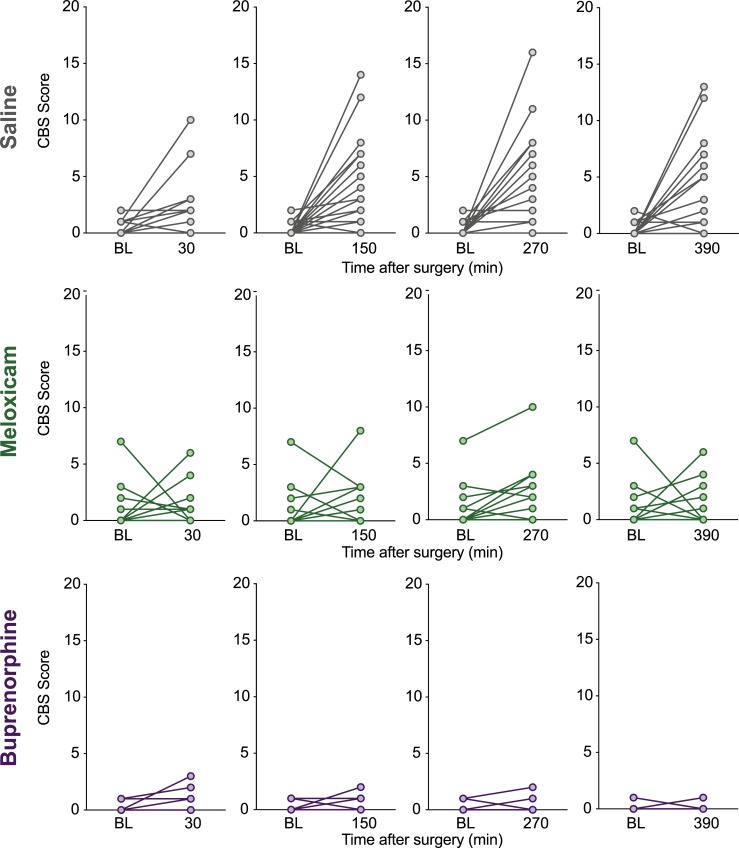
CBS scores of individual rats at all post-surgical timepoints in comparison to their baseline scores. Saline (n = 21), meloxicam (n = 16) and buprenorphine (n = 15) rats at all time points.

**Fig 5:**
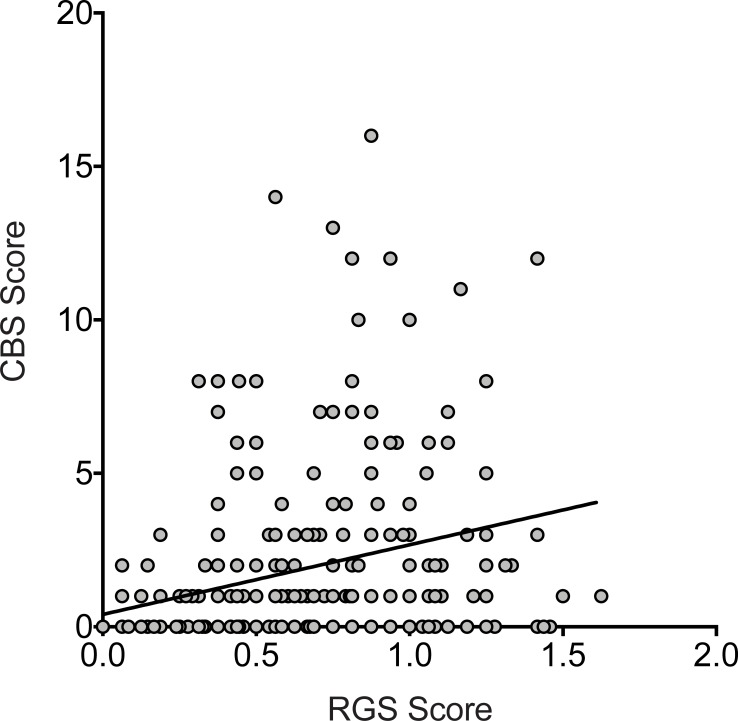
RGS and CBS scores are positively correlated. Data from all groups and all timepoints are plotted (260 xy pairs) with each point representing an individual rat. r = 0.36 (95% CI, 0.25 to 0.47), p < 0.0001.

Scores of individual animals at all post-surgical time points in comparison to their baseline scores are shown in Figs 3 and 4. Variability between individuals, quantified as a coefficient of variation, was generally greater with the CBS than the RGS (Table 3). RGS and CBS pain scores of the same rat were weakly positively correlated with one another (r = 0.36, 95% CI: 0.25 to 0.47, p < 0.0001, Fig 5).

**Table 3:** Coefficients of variation for RGS and CBS scores.

	RGS	CBS
Time point (m)	Coefficient of variation (%)	Coefficient of variation (%)
**Saline**		
BL	71.66	196.21
30	34.70	111.69
150	36.88	97.03
270	30.79	94.08
390	40.76	103.91
**Meloxicam**		
BL	65.08	212.28
30	32.93	115.37
150	54.81	127.01
270	35.34	121.98
390	50.88	107.47
**Buprenorphine**		
BL	60.65	263.90
30	36.93	151.71
150	52.56	185.16
270	60.26	222.61
390	71.42	263.90

## Discussion

The need for better measures to assess ongoing pain in laboratory rodents has prompted the development of two behavioural pain scales: the RGS and CBS. Both pain scales have demonstrated construct validity as scores increase in response to common pain models and decrease with analgesic administration [7–10]. However, these two scales have never been directly compared and the assessment of their strengths and weaknesses, specifically their use to assess the efficacy of different analgesics, has been limited. The RGS and CBS have produced conflicting reports on the efficacy of NSAIDs to treat post-laparotomy pain in rats [8–10, 13]. Therefore, this study compared the two scales using a laparotomy pain model and two different classes of analgesics.

Both the CBS and the RGS were effective in identifying postoperative pain, as the saline treated group displayed increased scores from baseline at all postsurgical timepoints. In the case of the RGS, postoperative pain scores exceeded a previously derived analgesic intervention threshold [11]. Pain scores for both scales were attenuated by buprenorphine which is consistent with the large body of evidence that buprenorphine offers effective post-operative pain relief [13, 16, 18]. In contrast to these negative (saline) and positive (buprenorphine) controls, treatment with meloxicam revealed a difference in the performance of the RGS and CBS. While CBS scores of meloxicam treated animals did not increase from baseline, the RGS scores of the same rats did so, to the extent that the RGS scores did not differ significantly from those of the saline group. Two possible interpretations arise from these data: 1) Meloxicam is an effective analgesic, as shown by the CBS, but the RGS is not sensitive enough to discriminate the decrease in pain, or 2) Meloxicam does not measurably alleviate post-surgical pain, as indicated by the RGS, and the CBS is less sensitive to differing pain levels.

The discrepancy between scales echoes previous studies where the CBS has displayed NSAIDs to be effective while the RGS reports questionable efficacy [8–10, 13]. Meloxicam at a dose of 2 mg/kg was shown to reduce CBS scores by approximately 50% (abnormal gait was also included in the score) following laparotomy [9]. Using the RGS as an outcome measure following a nerve root compression surgery, scores decreased in response to meloxicam (2 mg/kg) at 6 hours post-operatively compared to animals that did not receive analgesia [19]. However, the RGS scores in these animals were similar to those receiving sham surgery (hemilaminectomy without nerve compression), and these scores were greater than baseline and the scores of animals receiving anaesthesia alone. Thus, even though meloxicam reduced scores in the nerve root compression group, this reduction does not suggest that meloxicam provided sufficient analgesia. These results support the observations reported here but a direct comparison is limited by the difference in surgical model.

Similar observations have also been reported with two other NSAIDs: ketoprofen and carprofen. Low doses of these NSAIDs have been repeatedly reported to reduce CBS behaviours; however, these same NSAIDs were reported as either ineffective at reducing RGS scores or a much higher dose was required [8–10, 13, 20, 21]. For example, ketoprofen at 5 mg/kg significantly reduced the frequency of CBS behaviours but a higher dose of 25–40 mg/kg was required to reduce RGS scores significantly, a dose range associated with adverse gastrointestinal effects [8, 13, 20–23]. At high doses, ketoprofen is as effective as 0.8 mg/kg of morphine in reducing RGS scores [21].

The alternate interpretation for the apparent limited analgesic efficacy of meloxicam, as assessed by the RGS, is that the RGS is insensitive to its analgesic properties. The CBS and RGS were only weakly correlated to one another suggesting that they may measure different aspects of pain. An important limitation of pain assessment scales in animals is the underlying assumption that they are a true reflection of an animal’s experience. Methods that assess the impact of pain on broader aspects of rodent life such as sleep disturbances, social behaviour, and anxious behaviours have also shown mixed results as being reliable indicators of pain in different contexts [24–26]. To clarify purported analgesic effects and the sensitivity of different scales, one approach would be to use multiple measures and evaluate the totality of evidence [26, 27]. Such methods could include conditioned place preference testing or behaviours associated with well being [6, 28, 29]. Converging evidence from multiple pain assessment modalities will help to clarify results when discrepancies between methods arise, such as the one we identified here.

### Meloxicam vs buprenorphine

As discussed above, further work is needed to fully interpret the role of meloxicam as an analgesic for acute post-operative pain. Buprenorphine treatment reduced both CBS and RGS scores to baseline levels (and below a previously established intervention threshold) in this study and in others [9, 13, 16, 18]. Based on the RGS, buprenorphine is more effective in treating laparotomy pain. While the CBS does not discriminate between meloxicam and buprenorphine, the difference between saline and buprenorphine was greater than the difference in CBS scores between saline and meloxicam. This suggests that buprenorphine offers superior analgesia; however, the study was not powered to identify this magnitude of difference and the importance of such differences is unknown. Nevertheless, buprenorphine has limitations. It can cause increased activity and pica behaviour [30, 31]. As a result of these limitations and the shorter half-life compared to meloxicam (which necessitates more frequent dosing), NSAIDs remain a popular analgesic in rats [32–34].

### CBS vs RGS methodology

In examining RGS data from individual animals, following surgery almost all saline treated rats displayed RGS scores above the analgesic intervention threshold, while most buprenorphine treated rats fell below (Fig 3). Therefore, rats with a greater probability of being painful would receive analgesia, according to the RGS methodology. The CBS does not yet have an analgesic intervention threshold associated with it, thus it is difficult to know how much of an increase represents a meaningful increase in pain. The CBS also displayed greater variability and a handful of rats (some even in the saline treated group) did not show pain behaviours after surgery. In light of this, it is important to note that only three pain behaviours were included in the ethogram for this study. While our behaviours were chosen as they have been identified as important indicators of pain, adding more behaviours to the ethogram may reduce variability [10].

The CBS has been criticized as being labour intensive [13]; however, we were able to obtain scores with a 10 minute observation period and a 5 minute period has been reported [9]. In contrast to the RGS, the CBS has not been assessed for real-time observation [16]. This limitation along with the variability of the CBS data among individual animals suggests it may be better suited for a research setting where data are averaged. Limitations of the RGS are that raters may require more training [15], though recent advances in machine learning may eventually allow for fully automated scoring [35].

### Limitations

A limitation of this study was that only female rats were used. Males and females have been previously studied using both the CBS and the RGS, and sex differences were not identified [7, 8]. Sex differences in sensitivity to analgesics have been reported and this may have affected the results [32, 34, 36]. Therefore, these results should be generalized cautiously to males.

## Conclusions

This study provides direct evidence that the RGS and CBS differ with regards to interpreting meloxicam efficacy. Further investigation is required to assess the efficacy of meloxicam for acute post-operative pain in rats. It is likely that converging evidence from multiple outcome measures is required to achieve this.

## Supporting information

S1 Table Between strain comparisons of RGS and CBS scores of Sprague-Dawley and Wistar rats within treatment groups. Sprague-Dawley: saline: n = 11, meloxicam: n = 8, buprenorphine: n = 7; Wistar: saline: n = 10, meloxicam: n = 8, buprenorphine: n = 8. p values and 95% confidence intervals of the differences are reported for each timepoint.
(DOCX)

## References

[1] Backonja MM, Stacey B. Neuropathic pain symptoms relative to overall pain rating. J Pain. 2004;5(9):491–7. 10.1016/j.jpain.2004.09.001 15556827

[2] Bennett GJ. What Is Spontaneous Pain and Who Has It? J Pain. 2012;13(10):921–9. 10.1016/j.jpain.2012.05.008 22824584

[3] Mogil JS, Crager SE. What should we be measuring in behavioral studies of chronic pain in animals? Pain. 2004;112(1–2):12–5. 10.1016/j.pain.2004.09.028 15494180

[4] Mogil JS, Davis KD, Derbyshire SW. The necessity of animal models in pain research. Pain. 2010;151(1):12–7. 10.1016/j.pain.2010.07.015 20696526

[5] Rice ASC, Cimino-Brown D, Eisenach JC, Kontinen VK, Lacroix-Fralish ML, Machin I, et al Preclinical Pain C. Animal models and the prediction of efficacy in clinical trials of analgesic drugs: A critical appraisal and call for uniform reporting standards. Pain. 2008;139(2):243–7. 10.1016/j.pain.2008.08.017 18814968

[6] Roughan JV, Coulter CA, Flecknell PA, Thomas HD, Sufka KJ. The conditioned place preference test for assessing welfare consequences and potential refinements in a mouse bladder cancer model. Plos One. 2014;9(8):16. 10.1371/journal.pone.0103362 PMC4123882 25100208

[7] Sotocinal SG, Sorge RE, Zaloum A, Tuttle AH, Martin LJ, Wieskopf JS, et al The Rat Grimace Scale: A partially automated method for quantifying pain in the laboratory rat via facial expressions. Mol Pain. 2011;7:10 10.1186/1744-8069-7-10 21801409 PMC3163602

[8] Roughan JV, Flecknell PA. Behavioural effects of laparotomy and analgesic effects of ketoprofen and carprofen in rats. Pain. 2001;90(1–2):65–74. 11166971 10.1016/s0304-3959(00)00387-0

[9] Roughan JV, Flecknell PA. Evaluation of a short duration behaviour-based post-operative pain scoring system in rats. European Journal of Pain. 2003;7(5):397–406. 10.1016/S1090-3801(02)00140-4 12935791

[10] Roughan JV, Flecknell RA. Behaviour-based assessment of the duration of laparotomy-induced abdominal pain and the analgesic effects of carprofen and buprenorphine in rats. Behav Pharmacol. 2004;15(7):461–72. 15472568 10.1097/00008877-200411000-00002

[11] Oliver V, De Rantere D, Ritchie R, Chisholm J, Hecker KG, Pang DSJ. Psychometric assessment of the Rat Grimace Scale and development of an analgesic intervention score. Plos One. 2014;9(5):7. 10.1371/journal.pone.0097882 PMC4024023 24838111

[12] Roughan JV, Flecknell PA. Training in behaviour-based post-operative pain scoring in rats—An evaluation based on improved recognition of analgesic requirements. Applied Animal Behaviour Science. 2006;96(3–4):327–42.

[13] Waite ME, Tomkovich A, Quinn TL, Schumann AP, Dewberry LS, Totsch SK, et al Efficacy of common analgesics for postsurgical pain in rats. J Amer Assoc Lab Anim Sci. 2015;54(4):420–5. 26224443 PMC4521577

[14] Roughan JV, Flecknell PA. Buprenorphine: a reappraisal of its antinociceptive effects and therapeutic use in alleviating post-operative pain in animals. Lab Anim. 2002;36(3):322–43. 10.1258/002367702320162423 12144743

[15] Zhang EQ, Leung VSY, Pang DSJ. Influence of rater training on inter- and intrarater reliability when using the Rat Grimace Scale. J Amer Assoc Lab Anim Sci. 2019;58(2):178–83. 30755291 10.30802/AALAS-JAALAS-18-000044 PMC6433356

[16] Leung V, Zhang E, Pang DSJ. Real-time application of the Rat Grimace Scale as a welfare refinement in laboratory rats. Scientific Reports. 2016;6:12 10.1038/s41598-016-0010-7 27530823 PMC4987703

[17] Schuster CJ, Pang DSJ. Forced-air pre-warming prevents peri-anaesthetic hypothermia and shortens recovery in adult rats. Lab Anim. 2018;52(2):142–51. 10.1177/0023677217712539 28599579

[18] Guarnieri M, Brayton C, DeTolla L, Forbes-McBean N, Sarabia-Estrada R, Zadnik P. Safety and efficacy of buprenorphine for analgesia in laboratory mice and rats. Lab Anim. 2012;41(11):337–43. 10.1038/laban.152 23079917

[19] Philips BH, Weisshaar CL, Winkelstein BA. Use of the Rat Grimace Scale to evaluate neuropathic pain in a model of cervical radiculopathy. Comparative Med. 2017;67(1):34–42. PMC5310623 28222837

[20] Kawano T, Eguchi S, Iwata H, Yamanaka D, Tateiwa H, Locatelli FM, et al Effects and underlying mechanisms of endotoxemia on post-incisional pain in rats. Life Sci. 2016;148:145–53. 10.1016/j.lfs.2016.01.046 26835988

[21] Kawano T, Takahashi T, Iwata H, Morikawa A, Imori S, Waki S, et al Effects of ketoprofen for prevention of postoperative cognitive dysfunction in aged rats. J Anesth. 2014;28(6):932–6. 10.1007/s00540-014-1821-y 24676769

[22] Shientag LJ. Efficacy of common analgesics for postsurgical pain in rats. Journal of the American Association for Laboratory Animal Science: JAALAS. 2016;55(1):7. PMC4747002 26817971

[23] Shientag LJ, Wheeler SM, Garlick DS, Maranda LS. A therapeutic dose of ketoprofen causes acute gastrointestinal bleeding, erosions, and ulcers in rats. J Am Assoc Lab Anim Sci. 2012;51(6):832–41. 23294892 PMC3508190

[24] Monassi CR, Bandler R, Keay KA. A subpopulation of rats show social and sleep-waking changes typical of chronic neuropathic pain following peripheral nerve injury. European Journal of Neuroscience. 2003;17(9):1907–20. 12752790 10.1046/j.1460-9568.2003.02627.x

[25] Sheahan TD, Siuda ER, Bruchas MR, Shepherd AJ, Mohapatra DP, Gereau RW, et al Inflammation and nerve injury minimally affect mouse voluntary behaviors proposed as indicators of pain. Neurobiology of Pain. 2017;2:1–12. 10.1016/j.ynpai.2017.09.001 29075674 PMC5653321

[26] Urban R, Scherrer G, Goulding EH, Tecott LH, Basbaum AI. Behavioral indices of ongoing pain are largely unchanged in male mice with tissue or nerve injury-induced mechanical hypersensitivity. Pain. 2011;152(5):990–1000. 10.1016/j.pain.2010.12.003 21256675 PMC3079194

[27] Bateson P. Assessment of pain in animals. Anim Behav. 1991;42:827–39.

[28] Andrews N, Legg E, Lisak D, Issop Y, Richardson D, Harper S, et al Spontaneous burrowing behaviour in the rat is reduced by peripheral nerve injury or inflammation associated pain. European Journal of Pain. 2012;16(4):485–95. 10.1016/j.ejpain.2011.07.012 22396078

[29] Sufka KJ. Conditioned place preference paradigm—A novel approach for analgesic drug assessment against chronic pain. Pain. 1994;58(3):355–66. 7838585 10.1016/0304-3959(94)90130-9

[30] Allen M, Johnson RA. Evaluation of self-injurious behavior, thermal sensitivity, food intake, fecal output, and pica after injection of three buprenorphine formulations in rats (Rattus norvegicus). Am J Vet Res. 2018;79(7):697–703. 10.2460/ajvr.79.7.697 29943638

[31] Roughan JV, Flecknell PA. Effects of surgery and analgesic administration on spontaneous behaviour in singly housed rats. Res Vet Sci. 2000;69(3):283–8. 10.1053/rvsc.2000.0430 11124101

[32] Busch U, Schmid J, Heinzel G, Schmaus H, Baierl J, Huber C, et al Pharmacokinetics of meloxicam in animals and the relevance to humans. Drug Metabolism and Disposition. 1998;26(6):576–84. 9616195

[33] Gades NM, Danneman PJ, Wixson SK, Tolley EA. The magnitude and duration of the analgesic effect of morphine, butorphanol, and buprenorphine in rats and mice. Contemporary Topics in Laboratory Animal Science. 2000;39(2):8–13. 11487232

[34] Moody DE, Fang WFB, Morrison J, McCance-Katz E. Gender differences in pharmacokinetics of maintenance dosed buprenorphine. Drug and Alcohol Dependence. 2011;118(2–3):479–83. 10.1016/j.drugalcdep.2011.03.024 21515002 PMC3162987

[35] Tuttle AH, Molinaro MJ, Jethwa JF, Sotocinal SG, Prieto JC, Styner MA, et al A deep neural network to assess spontaneous pain from mouse facial expressions. Mol Pain. 2018;14. 10.1177/1744806918763658 PMC5858615 29546805

[36] Craft RM, Mogil JS, Aloisi AM. Sex differences in pain and analgesia: the role of gonadal hormones. European Journal of Pain. 2004;8(5):397–411. 10.1016/j.ejpain.2004.01.003 15324772

