# Supplementary material for: Comparing the Rat Grimace Scale and a composite behaviour score in rats
Source: PLoS One. 2019 May 31;14(5):e0209467. doi: 10.1371/journal.pone.0209467 (PMC6544219; doi:10.1371/journal.pone.0209467)
Supplement: S1 Table — Sprague-Dawley: saline: n = 11, meloxicam: n = 8, buprenorphine: n = 7; Wistar: saline: n = 10, meloxicam: n = 8, buprenorphine: n = 8. p values and 95% confidence intervals of the differences are reported for each timepoint. (DOCX) [file pone.0209467.s001.docx]

|  | **RGS** | **CBS** |
| --- | --- | --- |
| Comparison | p-value [95% CI] | p-value [95% CI] |
| **Saline** | | |
| BL | 0.93 [-0.42 to 0.22] | > 0.99 [-3.9 to 4.0] |
| 30 min | > 0.99 [-0.28 to 0.35] | 0.96 [-2.9 to 5.0] |
| 150 min | > 0.99 [-0.37 to 0.27] | > 0.99 [-4.5 to 3.4] |
| 270 min | 0.62 [-0.15 to 0.48] | > 0.99 [-4.6 to 3.3] |
| 390 min | > 0.99 [-0.31 to 0.32] | 0.51 [-6.2 to 1.7] |
| **Meloxicam** | | |
| BL | 0.78 [-0.54 to 0.22] | 0.88 [-1.8 to 3.8] |
| 30 min | 0.14 [-0.70 to 0.061] | 0.74 [-4.0 to 1.5] |
| 150 min | 0.46 [-0.61 to 0.15] | 0.65 [-1.4 to 4.1] |
| 270 min | > 0.99 [-0.39 to 0.37] | 0.92 [-1.9 to 3.6] |
| 390 min | 0.81 [-0.53 to 0.22] | 0.40 [-4.5 to 1.0] |
| **Buprenorphine** | | |
| BL | 0.69 [-0.54 to 0.19] | 0.94 [-0.59 to 1.1] |
| 30 min | 0.96 [-0.26 to 0.46] | 0.85 [-0.52 to 1.2] |
| 150 min | > 0.99 [-0.34 to 0.38] | 0.99 [-1.0 to 0.66] |
| 270 min | > 0.99 [-0.40 to 0.32] | > 0.99 [-0.88 to 0.81] |
| 390 min | 0.513 [-0.57 to 0.15] | > 0.99 [-0.86 to 0.82] |

**S1 Table. Between strain comparisons of RGS and CBS scores of Sprague-Dawley and Wistar rats within treatment groups**. Sprague-Dawley: saline: n = 11, meloxicam: n = 8, buprenorphine: n = 7; Wistar: saline: n = 10, meloxicam: n = 8, buprenorphine: n = 8. p values and 95% confidence intervals of the differences are reported for each timepoint.
